# Nanga Parbat – The mountain of destiny

**DOI:** 10.1113/EP092562

**Published:** 2025-02-07

**Authors:** Peter Bärtsch

**Affiliations:** ^1^ Department of Internal Medicine, Division of Sports Medicine University Clinic Heidelberg Heidelberg Germany

Nanga Parbat is 8125 m high, the ninth highest mountain of the world, and known as the Mountain of Destiny for German mountaineers. It is located at the western end of the Himalayas and easily accessible by short approaches from Pakistan and Kashmir. Because the Kingdom of Nepal was closed to foreigners until 1950, Nanga Parbat was the best option for climbing one of the 14 mountains higher than 8000 m. Between 1932 and 1939 five German expeditions tried it but did not get above 7850 m on the northern side of Nanga Parbat. A total of 25 people lost their lives in heavy snowstorms and ice avalanches. After the Second World War, Karl Maria Herrligkofer, a medical doctor in Munich, whose half‐brother had died in the 1934 expedition on Nanga Parbat, led eight further expeditions to this mountain. This resulted in the first ascents of three different routes by distinguished climbers: Hermann Buhl in 1953 from the north via Rakhiot Peak, Toni Kinshofer and colleagues from the west (Diamir Face in 1970), and Reinhold and Günter Messner from the south (Rupal face in 1970). By 1978, 19 mountaineers from nine expeditions had reached the summit and 36 mountaineers had died (Messner, 1981).

In 1984 I had finished my training in Internal Medicine and was free to join a Swiss expedition to Nanga Parbat over the Kinshofer route on the Diamir face. The core team consisted of five renowned mountaineers, who had all climbed the north faces of the Eiger and Matterhorn. Three of them had also reached the summit of other 8000 m peaks prior, while my highest peaks were volcanoes in South America up to 6400 m only. I was interested in high altitude medicine, and had investigated how exercise impacts haemostasis on the Jungfraujoch (3450 m) (Bärtsch et al., 1982). I was excited about getting involved in a big adventure, but, with the history on Nanga Parbat in mind, I was also concerned about the medical challenges as well as the climbing challenges I might face given my lack of experience at extreme altitudes.

Starting in late April at 1400 m in the Indus Valley, it took only 4 days over an exposed path along steep canyon cliffs for our team of seven climbers to reach base camp at 4000 m. Sixty‐eight porters carried our equipment and left us all by ourselves for 7 weeks on a snow‐covered meadow in front of the Diamir face together with a liaison officer and a local cook. We were in splendid isolation with a challenging goal. A mail runner between base camp and the post office of Chilas in the Indus valley provided the only connection to the ‘outside’ world twice a week. A fairly slow ascent rate of about 650 m per day and pre‐acclimatization by ski touring in the Alps prevented clinically relevant symptoms of acute mountain sickness (AMS) after arrival at base camp (Bärtsch & Swenson, 2013).

AMS is characterized by non‐specific symptoms like headache, loss of appetite or nausea, dizziness, and fatigue that occur after rapid exposure to altitudes above 2500 m (Luks et al., 2024). The major risk factors are fast ascent to altitudes above 3500 m and increased individual susceptibility. The major treatment is analgesics and rest days, or descent if symptoms persist. Inappropriate management of AMS can lead to potentially lethal high altitude pulmonary (HAPE) or cerebral (HACE) oedema for which immediate descent or supplemental oxygen in addition to treatment with drugs (pulmonary vasodilators for HAPE and dexamethasone for HACE) can be lifesaving. Since at the time ascent profiles of expeditions (see Figure 1) allowed sufficient time for acclimatization and increased individual susceptibility could be excluded based on the mountaineering history, AMS, HAPE or HACE were rare. Moreover, early treatment of AMS was usually appropriate and prevented development of HACE or HAPE.

**FIGURE 1:**
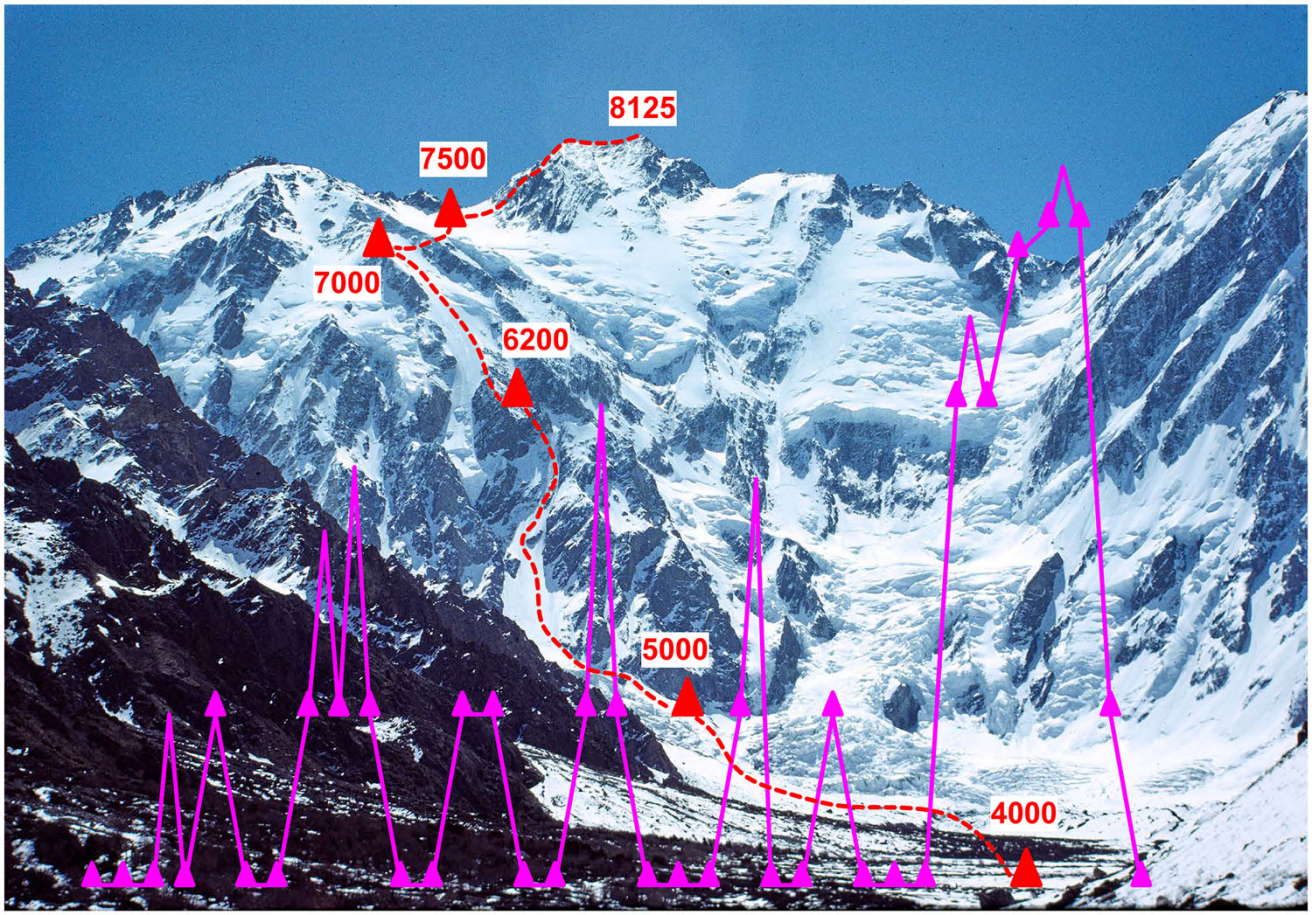
Diamir face of Nanga Parbat with ascent route, location and altitude (in meters) of camps shown in red. The pink lines show the ascent profile of the author with triangles indicating overnight stays (Photo Peter Bärtsch).

I still remember vividly the startling moment just before arriving at base camp when a 4000 m‐high ice wall suddenly appeared around the corner only a few kilometres away (Figure 1). It was hard to imagine that we would be able to climb at 8000 m when we already felt a considerable impairment of performance at base camp as our arterial partial pressure of oxygen ($P_{O_{2}}$) and arterial oxyhaemoglobin saturation ($S_{aO_{2}}$) must have been in the order of 40 mmHg and 80%, respectively, as suggested by blood gas analysis measured in mountaineers without AMS on the day of arrival at 4559 m in the Alps (Bartsch et al., 1987).

Indeed, it was known that sudden exposure to 8000 m would lead to a loss of consciousness within about 5 min (Izraeli et al., 1988) while we could expect to have a maximal aerobic performance of about 100 W at 8000 m after acclimatizing on the mountain over a month (Sutton et al., 1988). This huge gain of performance is achieved by moving up and down on the mountain setting up fixed ropes and camps as shown in the ascent profile we adopted (Figure 1), which was standard practice at the time.

We had two teams that took turns in working on the mountain and recovering at base camp. With each new turn on the mountain, I noticed a remarkable increase in submaximal performance. The major factors accounting for an improved performance with prolonged stay at high altitude are increased erythropoiesis and reduction of plasma volume (Siebenmann et al., 2017) as well as ventilatory acclimatization due to increased sensitivity of the peripheral chemoreceptors (Sato et al., 1992) and shifting of the hypercapnic ventilatory response to a lower arterial partial pressure of carbon dioxide ($P_{\mathrm{aC}O_{2}}$) (Kellogg, 1963). After several weeks at 5300 m, haemoglobin concentration in the blood increases from 14 to 18.5 g%, and increased ventilation lowers $P_{\mathrm{aC}O_{2}}$ from 27 to 21 mmHg and increases $S_{aO_{2}}$ from 63 to 75% (Calbet et al., 2003). Thus, arterial O_2_ content of 1 L blood increases from 118 to 186 mL, since 1 g fully saturated haemoglobin binds 1.34 mL O_2_. Thus, acclimatization at 5300 m increases arterial O_2_ content by 57%, accounting for improved submaximal performance. Nevertheless, maximal aerobic performance decreases by about 1% per 100 m above 1500 m (Fulco & Cymerman, 1998) and is not affected by acclimatization (Calbet et al., 2003).

In the first 2 days after arrival at 4000 m we rested, set up base camp and explored the new environment. Then we began carrying equipment to camp 1 at 5000 m on skis, which minimized the effort of climbing in deep snow and made descent a pleasure. After having spent two nights at camp 1, we felt ready to install a fixed rope through the exposed ice channel or couloir to camp 2 at 6200 m (Figure 2). This work was delayed several times by heavy wind and snowfall. Eleven days later, I arrived for the first time at camp 2 just to find that the storm had destroyed the tent, which meant that descending in the couloir under great risk of rock fall in the afternoon was our only option. Two further attempts to reach the since‐restored camp 2 were blocked by bad weather and snowfall again.

**FIGURE 2:**
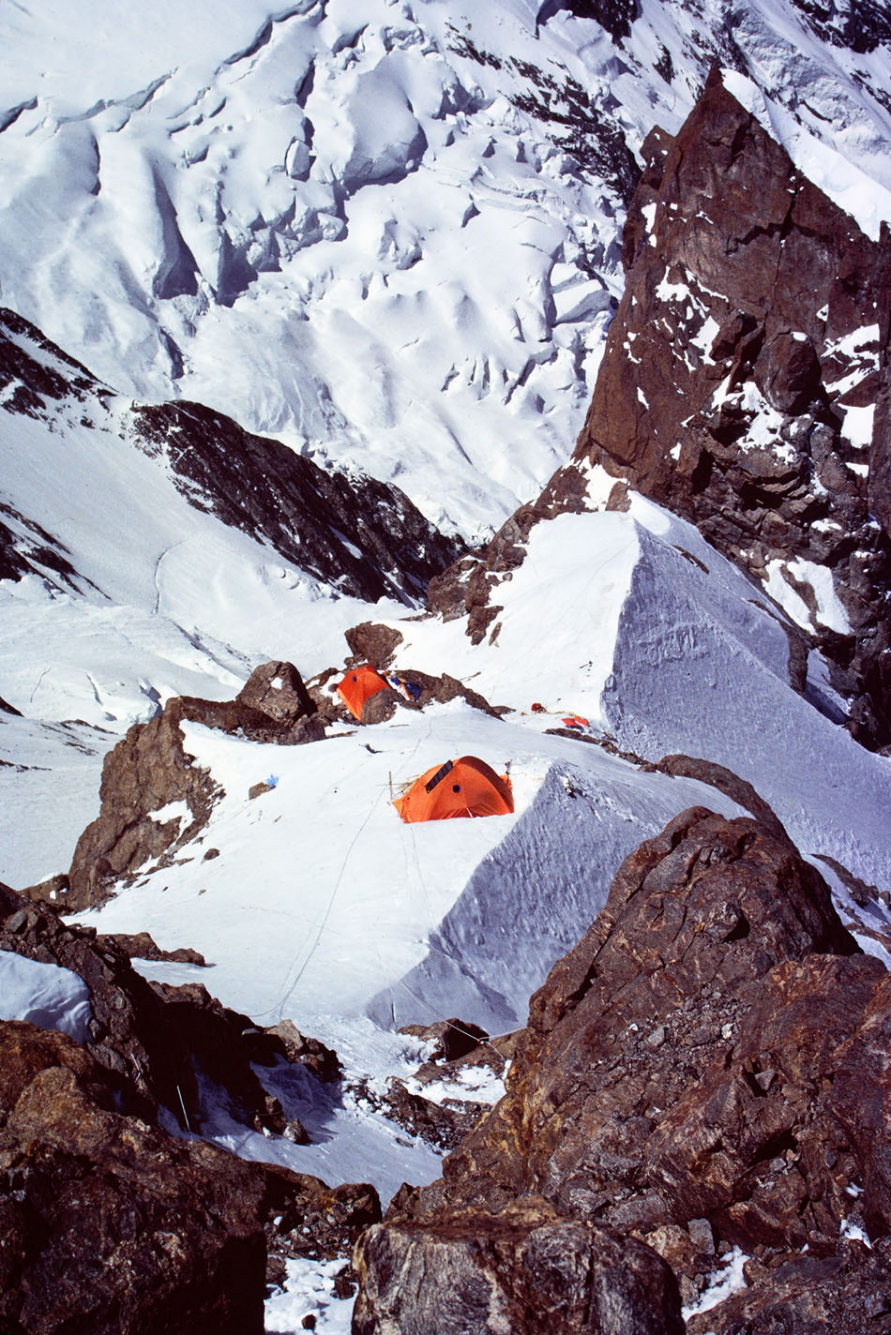
Camp 2 located at a ridge at 6200 m (Photo Peter Bärtsch).

Thus, unfavourable weather and snow conditions had prevented us from going beyond 6200 m for 2 weeks after we had originally set up camp 2. Nevertheless, we acclimatized well to the altitude, feeling ‘at home’ in camp 1. A steadily increasing submaximal performance made advancing up the couloir easier with each climb. It was also essential to have ample and palatable food at base camp for refuelling since weight loss occurs above 5000 m (Boyer & Blume, 1984; Pugh, 1962) due to less appetite and lower food palatability (Westerterp‐Plantenga et al., 1999).

Finally, good weather allowed returning in 1 day from base camp to camp 2 to continue the ascent. To our surprise, we discovered that fixed ropes above camp 2 mounted by an expedition in the previous year were still usable. This was essential for a rapid and safe climb through the Kinshofer icefield to camp 3 at 7000 m (Figure 3) and for crossing the steep slope leading to the Bazin glacier basin, where we set up camp 4 at about 7500 m (Figure 5). Thus, we could compensate for the delay caused by bad weather and were ready to take our chance to reach the summit just in time. We reasoned that a fairly good acclimatization to the altitude of 6000 m would allow for a rapid dash to the summit in 3 days. This tactic worked for three climbers of the summit team while one had to descend along the fixed rope for recovery because he had not recovered from AMS at camp 3 overnight.

**FIGURE 3:**
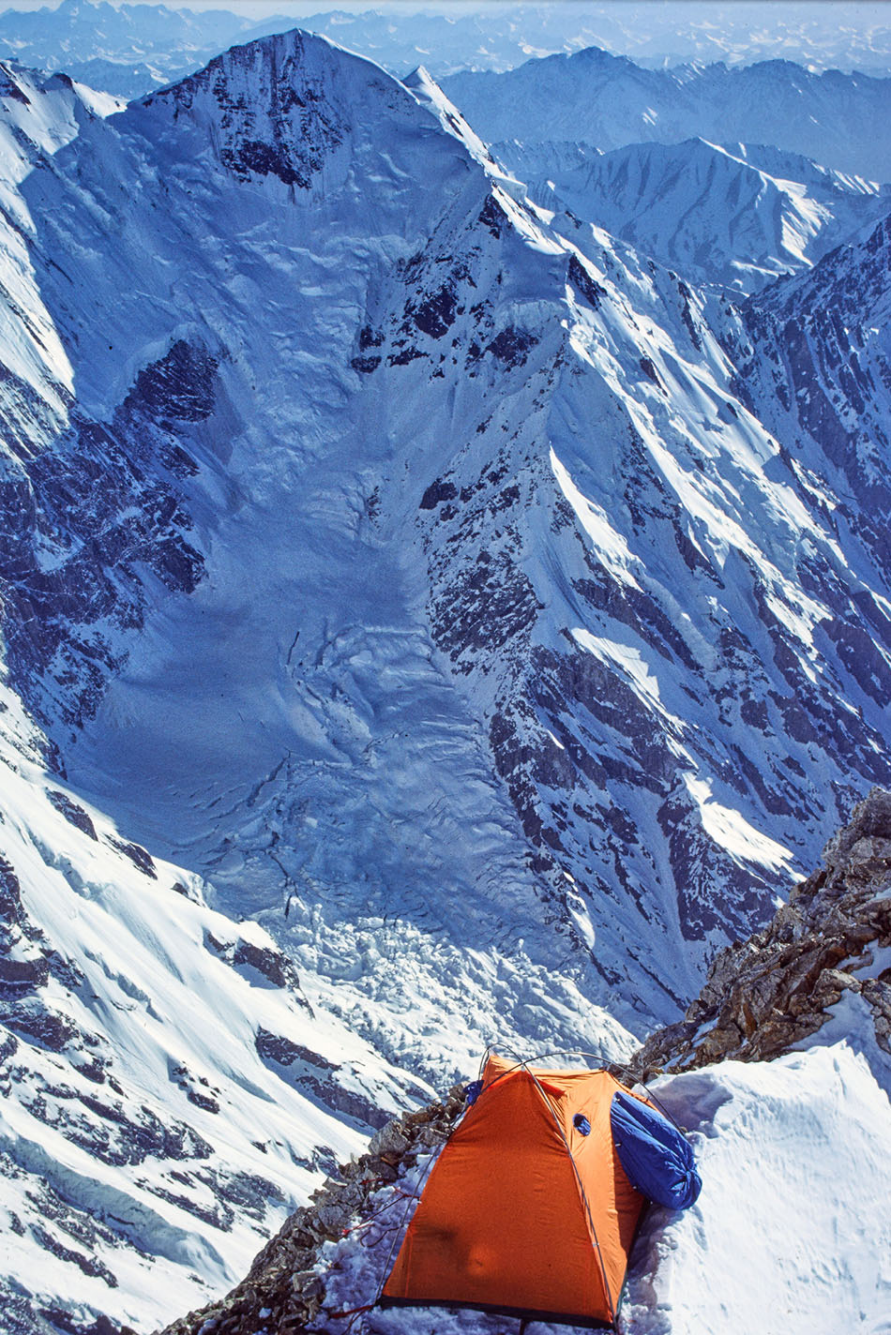
Exposed site of camp 3 with view to the Southern end of the Himalayas in Pakistan behind the Mazeno Ridge (Photo Peter Bärtsch).

The impact of hypoxia at these altitudes became more challenging. Sleep was less restful because of more central apnoeas due to periodic breathing, which increases with altitude (Anholm et al., 1992) and with ventilatory acclimatization (Bloch et al., 2010). Furthermore, the maximal aerobic capacity is decreased by about 60% at 7500 m (Fulco & Cymerman, 1998). For me (70 kg, ${\dot{V}}_{O_{2}\max}$ 55 mL/kg/min at sea level) this meant a ${\dot{V}}_{O_{2}\max}$ based on body weight was only 22 mL/kg/min. My rucksack with the equipment necessary for the summit attempt weighed about 15 kg (Figure 4). Assuming that the altitude‐induced weight loss equalled the weight of the clothing and gear carried on the body, the weight of my rucksack reduced my ${\dot{V}}_{O_{2}\max}$ to about 18 mL/kg/min when approaching camp 4. Such values are measured near sea level in patients with heart failure (Roibal Pravio et al., 2021) in whom pump failure due to heart disease accounts for the marginal oxygen supply to the body, while it was shown that cardiac function in healthy people is not impaired at the simulated altitude of Mount Everest (8848 m) (Reeves et al., 1987).

**FIGURE 4:**
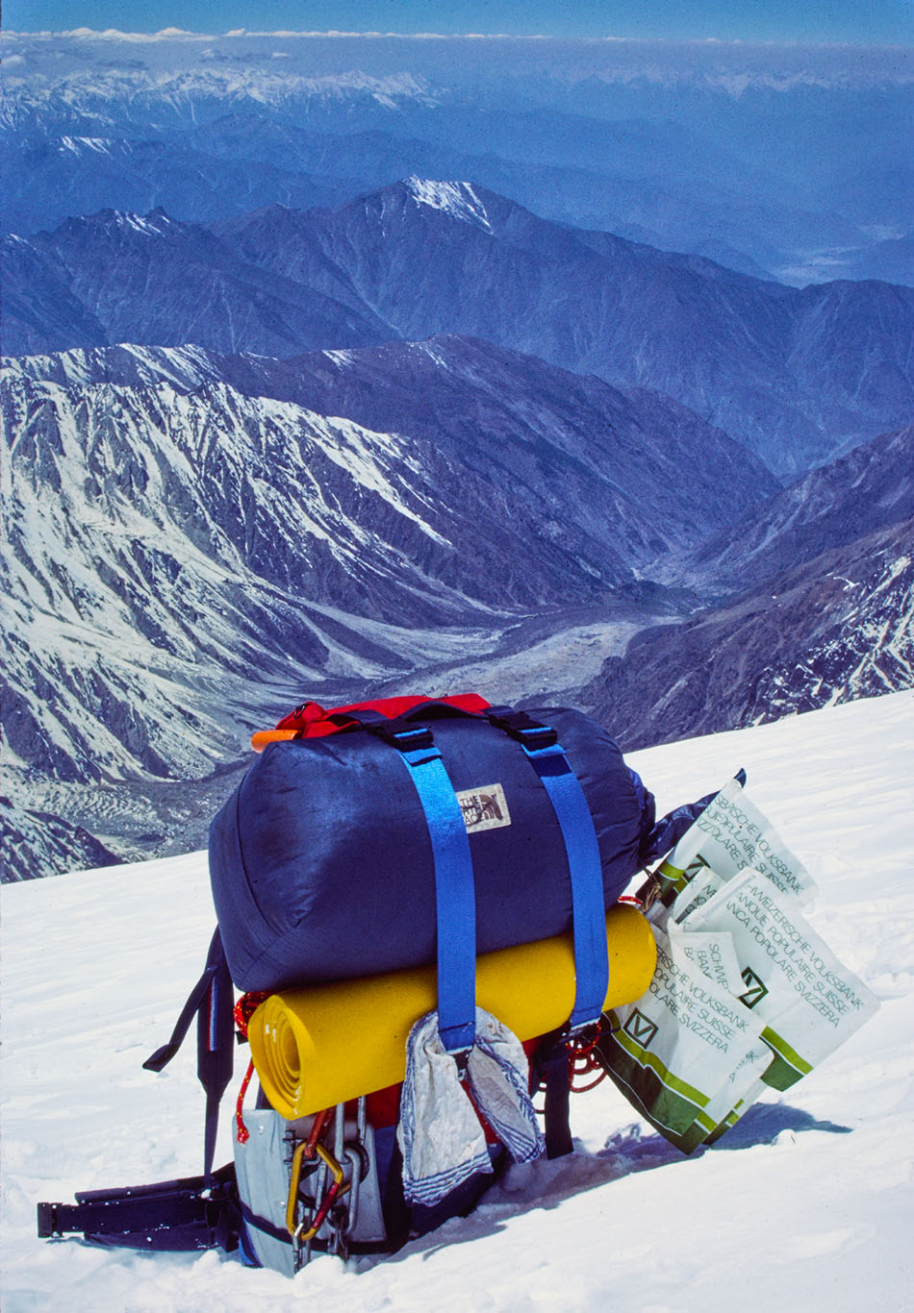
Rucksack of the author in the Bazin glacier basin. View down toward base camp, which is located on the meadow at the right side of the Diamir glacier and toward the Indus valley 6000 m below (Photo Peter Bärtsch).

Performance of heart failure patients is clinically characterized by the number of stair flights they can climb continuously. Thus, climbing toward camp 4 in the Bazin glacier base, I felt like a heart failure patient. Advancing in a stead state was impossible. I assume to have climbed close to my actual ${\dot{V}}_{O_{2}\max}$. Two ski poles helped to minimize the oxygen consumption for keeping my balance. My goal was to go 50 steps in a row (equivalent to about five stair flights), but often I was hanging on the ski poles for recovery earlier. Sitting down was no option because the effort of getting up again would have wiped out all the benefits of resting. If you are not ultimately determined to reach the summit of the mountain, you wonder what this is all about and what you have to prove to yourself. While I was sitting outside the tent of camp 4 at the foot of the summit pyramid of Nanga Parbat (Figure 5) with a cup of tea, these demotivating thoughts were blown away by the fascinating view from the top of the world down to the Indus River 6000 m below and to the snow‐covered Hindukush mountains of Afghanistan at the horizon.

**FIGURE 5:**
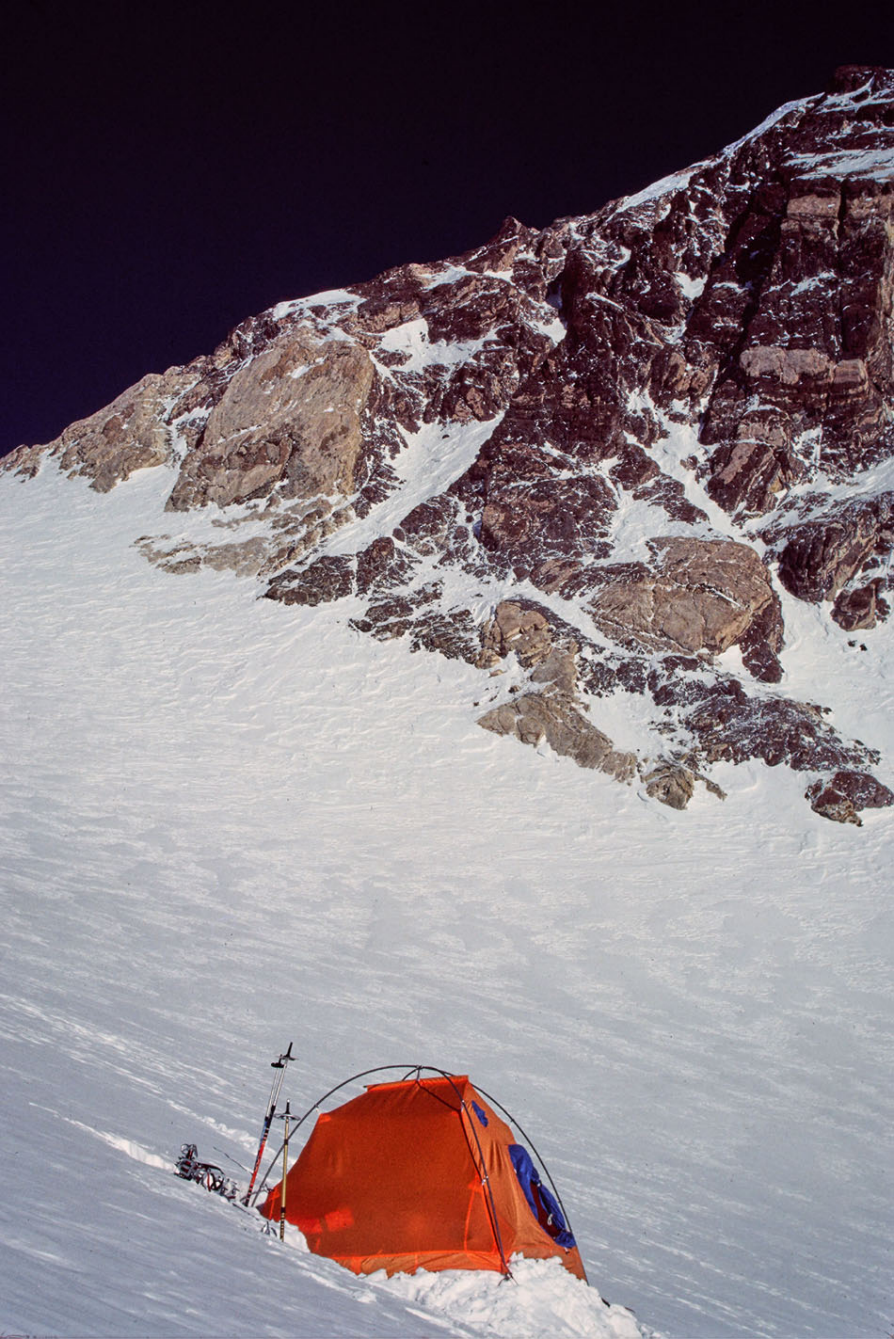
Camp 4 at 7500 m with  the lower, part of the couloir ascending to the right and leading to the top of the summit pyramid (Photo Peter Bärtsch).

On the next day, Fredy Graf, the organizer of the expedition, Marcel Rüedi, the most prominent climber of the team, and I set out for the summit of Nanga Parbat though a 45–50° steep ice channel. The conditions were perfect with a clear sky, no wind and good grip on the ice. Each of us climbed at his own speed. Carrying only a day pack improved my climbing performance greatly during the first hours. As it got steeper and higher, my concerns grew that I was climbing—not roped—beyond my limits. Despite a ventilation of about 90 L/min for 60 W resulting in a $P_{aO_{2}}$ of 33 mmHg, $P_{\mathrm{aC}O_{2}}$ of 13 mm Hg and $S_{aO_{2}}$ of 62% (Sutton et al., 1988), my brain analysed the situation correctly, reminding me that fatigue increases with time, climbing down is more difficult than climbing up, and that a climber of the Kinshofer expedition fell to his death in this couloir on the way down. My subsequent decision to turn around at about 7900 m was reinforced by Fredy, climbing slightly below me (Figure 6), who had early frostbite in his fingers, likely due to insufficient fluid intake caused by the nausea associated with mild AMS.

**FIGURE 6:**
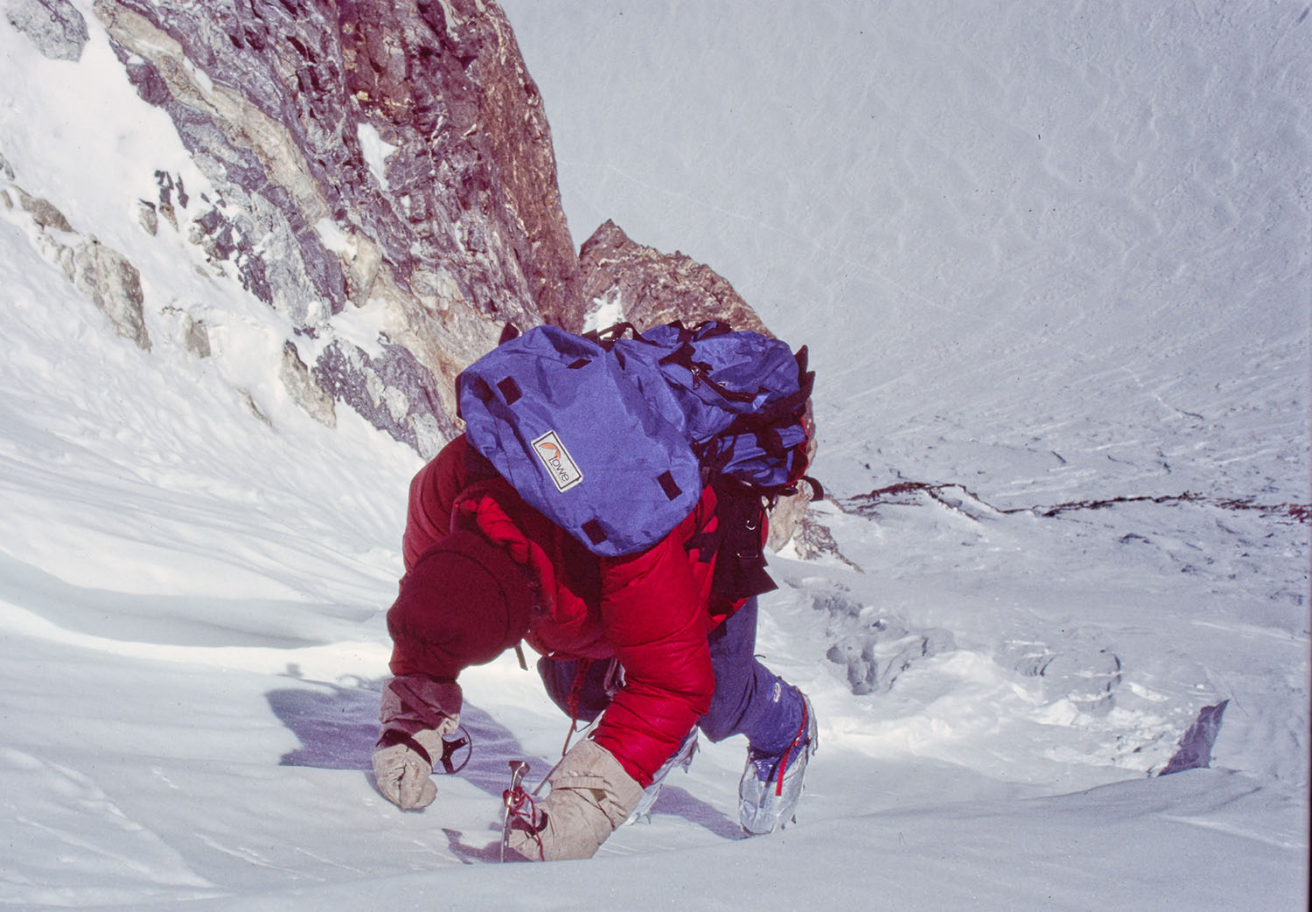
Climbing partner of the author at about 7900 m in the couloir leading to the summit. Note that the front teeth of the crampons have a good grip. We were not roped and had no second anchor instrument besides the ice ax (Photo Peter Bärtsch).

Thus, we turned around while Marcel was already far ahead on the way to his fifth successful climb of an ‘8000 m peak’. Fredy descended to camp 2 for recovery while I waited in camp 4 for Marcel, who returned in the afternoon. The next morning, we descended in fog and wind after a heavy snowfall at night. Fortunately, with the help of ‘invisible’ yet fluttering paper flags advertising in white and green for a Swiss bank, we found the beginning of the fixed rope—the lifeline for traversing the steep slope loaded with fresh snow in which we luckily did not trigger an avalanche.

Our experience shows that healthy trained mountaineers can climb ‘low’, 8000 m peaks without the help of supplemental oxygen and high‐altitude porters, if they take sufficient time for acclimatization. Accordingly, supplemental oxygen was not considered necessary at the time except for climbing Mt Everest and perhaps K2. The analysis of 92 deaths on Mt Everest between 1982 and 2006 showed that the vast majority occurred above 8000 m and mostly during descent. The average mortality rate was about 2.5% (Firth et al., 2008). Supplemental oxygen usually used above 8000 m with a flowmeter set at 2 L/min reduced the overall mortality on Mt Everest 2.7‐fold (Huey & Eguskitza, 2000). Nowadays, supplemental oxygen is also used in commercial expeditions to popular low 8000 m peaks such as Cho Oyu. Furthermore, the altitude for using supplemental oxygen on Mt Everest today appears to have decreased to 7300 m on the south side (Sherpa et al., 2024).

Regarding the use of supplemental oxygen, one has to consider that the flowmeters are not calibrated to the hypobaric environment at which they are used. Therefore, the effective oxygen flow above 8000 m is about 2–4 times higher (depending on the altitude and type of regulator) than indicated on the flow meter. A nominal flow of 2 L/min at an altitude of 8100 m increases $S_{pO_{2}}$ to values measured in ambient air at 3600 m and on top of Mt. Everest to those measured at 5000 m (Wakeham D. and Levine BD. et al., personal communication). Thus, supplemental oxygen is a game changer, which may save lives at extreme altitudes but is not necessary for climbing low, 8000 m peaks. Companies that provide supplemental oxygen on Mt Everest already above 6000 m and a flow of oxygen of 6–8 L/min on the summit day downgrade this mountain to a 4000 m peak at the most.

The Swiss Nanga Parbat 1984 Expedition was the highlight of my mountaineering career. Moreover, as it turned out, Nanga Parbat also became my Mountain of Destiny. Preparing the medical equipment for the expedition, I sought advice from Dr Oswald Oelz, an experienced expedition doctor, who had climbed Mt Everest. He had read my coagulation paper (Bärtsch et al., 1982) and was planning to study the epidemiology of AMS in the Margherita hut at 4559 m (Maggiorini et al., 1990). I was excited about joining his team after returning from Nanga Parbat and found signs of increased in vivo fibrin formation in four mountaineers with advanced HAPE (Bartsch et al., 1987). This result led to the question whether fibrin formation was a cause or a consequence of HAPE. Since epidemiological studies in Peru suggested that the recurrence of HAPE after a first episode is high (Hultgren & Marticorena, 1978), I performed a prospective study on mountaineers with a history of HAPE, which showed that fibrin formation was not a cause of HAPE (Bärtsch et al., 1989). This study design became the basis for further studies on all aspects of HAPE in the Margherita hut located at 4559 m in the Swiss Italian Alps. It also led to a long‐term employment for me in the research laboratory of Prof. P. W. Straub, the head of Internal Medicine at the Inselspital in Bern, and finally to the Chair of Sports Medicine at the University of Heidelberg.

## AUTHOR CONTRIBUTIONS

Sole author.

## CONFLICT OF INTEREST

None declared.

## FUNDING INFORMATION

No funding was received for this work.

## References

[eph13758-bib-0001] Anholm, J. D. , Powles, A. C. P. , Downey, R. , Houston, C. S. , Sutton, J. R. , Bonnet, M. H. , & Cymerman, A. (1992). Operation Everest II: Arterial oxygen saturation and sleep at extreme simulated altitude. American Review of Respiratory Disease, 145(4_pt_1), 817–826. 1554208 10.1164/ajrccm/145.4_Pt_1.817

[eph13758-bib-0002] Bärtsch, P. , Haeberli, A. , Franciolli, M. , Kruithof, E. K. O. , & Straub, P. W. (1989). Coagulation and fibrinolysis in acute mountain sickness and beginning pulmonary edema. Journal of Applied Physiology, 66(5), 2136–2144. 2745282 10.1152/jappl.1989.66.5.2136

[eph13758-bib-0003] Bärtsch, P. , Schmidt, E. K. , & Straub, P. W. (1982). Fibrinopeptide A after strenuous physical exercise at high altitude. Journal of Applied Physiology, 53(1), 40–43. 6811525 10.1152/jappl.1982.53.1.40

[eph13758-bib-0004] Bärtsch, P. , & Swenson, E. R. (2013). Clinical practice: Acute high‐altitude illnesses. New England Journal of Medicine, 368(24), 2294–2302. 23758234 10.1056/NEJMcp1214870

[eph13758-bib-0005] Bartsch, P. , Waber, U. , Haeberli, A. , Maggiorini, M. , Kriemler, S. , Oelz, O. , & Straub, W. P. (1987). Enhanced fibrin formation in high‐altitude pulmonary edema. Journal of Applied Physiology, 63(2), 752–757. 3654438 10.1152/jappl.1987.63.2.752

[eph13758-bib-0006] Bloch, K. E. , Latshang, T. D. , Turk, A. J. , Hess, T. , Hefti, U. , Merz, T. M. , Bosch, M. M. , Barthelmes, D. , Hefti, J. P. , Maggiorini, M. , & Schoch, O. D. (2010). Nocturnal periodic breathing during acclimatization at very high altitude at Mt. Muztagh Ata (7546 m). American Journal of Respiratory and Critical Care Medicine, 182(4), 562–568. 20442435 10.1164/rccm.200911-1694OC

[eph13758-bib-0007] Boyer, S. J. , & Blume, F. D. (1984). Weight loss and changes in body composition at high altitude. Journal of Applied Physiology, 57(5), 1580–1585. 6520055 10.1152/jappl.1984.57.5.1580

[eph13758-bib-0008] Calbet, J. A. , Boushel, R. , Radegran, G. , Sondergaard, H. , Wagner, P. D. , & Saltin, B. (2003). Why is VO_2 max_ after altitude acclimatization still reduced despite normalization of arterial O_2_ content?. American Journal of Physiology‐Regulatory, Integrative and Comparative Physiology, 284(2), R304–R316. 12388462 10.1152/ajpregu.00156.2002

[eph13758-bib-0009] Firth, P. G. , Zheng, H. , Windsor, J. S. , Sutherland, A. I. , Imray, C. H. , Moore, G. W. K. , Semple, J. L. , Roach, R. C. , & Salisbury, R. A. (2008). Mortality on Mount Everest, 1921–2006: Descriptive study. British Medical Journal, 337(dec11 1), a2654. 19074222 10.1136/bmj.a2654 PMC2602730

[eph13758-bib-0010] Fulco, C. S. , & Cymerman, A. (1998). Maximal and submaximal exercise performance at altitude. Aviation Space and Environmental Medicine, 69(8), 793–801. 9715971

[eph13758-bib-0011] Huey, R. B. , & Eguskitza, X. (2000). Supplemental oxygen and mountaineer death rates on Everest and K2 (Letter). The Journal of the American Medical Association, 284(2), 181. 10889590 10.1001/jama.284.2.181-a

[eph13758-bib-0012] Hultgren, H. N. , & Marticorena, E. A. (1978). High altitude pulmonary edema. Epidemiologic observations in Peru. Chest, 74(4), 372–376. 699645 10.1378/chest.74.4.372

[eph13758-bib-0013] Izraeli, S. , Avgar, D. , Glikson, M. , Shochat, I. , Glovinsky, Y. , & Ribak, J. (1988). Determination of the “time of useful consciousness” (TUC) in repeated exposures to simulated altitude of 25,000 ft (7,620 m). Aviation Space and Environmental Medicine, 59, (11 Pt 1), 1103–1105. 3202796

[eph13758-bib-0014] Kellogg, R. H. (1963). The role of CO2 in altitude acclimatization. In D. J. C. Cunningham (Ed.), The regulation of human respiration (pp. 379–394). Blackwell Scientific Publications.

[eph13758-bib-0015] Luks, A. M. , Beidleman, B. A. , Freer, L. , Grissom, C. K. , Keyes, L. E. , McIntosh, S. E. , Rodway, G. W. , Schoene, R. B. , Zafren, K. , & Hackett, P. H. (2024). Wilderness medical society clinical practice guidelines for the prevention, diagnosis, and treatment of acute altitude illness: 2024 update. Wilderness & Environmental Medicine, 35, (1_suppl), 2S–19S. 37833187 10.1016/j.wem.2023.05.013

[eph13758-bib-0016] Maggiorini, M. , Bühler, B. , Walter, M. , & Oelz, O. (1990). Prevalence of acute mountain sickness in the Swiss Alps. British Medical Journal, 301(6756), 853–855. 2282425 10.1136/bmj.301.6756.853 PMC1663993

[eph13758-bib-0017] Messner, R. (1981). Solo: Nanga Parbat. (1st ed.). Oxford University Press.

[eph13758-bib-0018] Pugh, L. G. (1962). Physiological and medical aspects of the Himalayan scientific and mountaineering expedition, 1960–1961. British Medical Journal, 2(5305), 621–627. 14489161 10.1136/bmj.2.5305.621 PMC1925967

[eph13758-bib-0019] Reeves, J. T. , Groves, B. M. , Sutton, J. R. , Wagner, P. D. , Cymerman, A. , Malconian, M. K. , Rock, P. B. , Young, P. M. , & Houston, C. S. (1987). Operation Everest II: Preservation of cardiac function at extreme altitude. Journal of Applied Physiology, 63(2), 531–539. 3654411 10.1152/jappl.1987.63.2.531

[eph13758-bib-0020] Roibal Pravio, J. , Barge Caballero, E. , Barbeito Caamaño, C. , Paniagua Martin, M. J. , Barge Caballero, G. , Couto Mallon, D. , Pardo Martinez, P. , Grille Cancela, Z. , Blanco Canosa, P. , García Pinilla, J. M. , Vázquez Rodríguez, J. M. , & Crespo Leiro, M. G. (2021). Determinants of maximal oxygen uptake in patients with heart failure. ESC Heart Failure, 8(3), 2002–2008. 33773098 10.1002/ehf2.13275 PMC8120347

[eph13758-bib-0021] Sato, M. , Severinghaus, J. W. , Powell, F. L. , Xu, F.‐D. , & Spellman, M. J. (1992). Augmented hypoxic ventilatory response in men at altitude. Journal of Applied Physiology, 73(1), 101–107. 1506356 10.1152/jappl.1992.73.1.101

[eph13758-bib-0022] Sherpa, K. , Sherpa, P. P. , Sherpa, T. , Rothenbühler, M. , Ryffel, C. , Sherpa, D. , Sherpa, D. R. , Sherchand, O. , Galuszka, O. , Dernektsi, C. , Reichlin, T. , & Pilgrim, T. (2024). Risk of cardiac arrhythmias among climbers on Mount Everest. The Journal of the American Medical Association Cardiology, 9(5), 480–485. 38568602 10.1001/jamacardio.2024.0364 PMC10993151

[eph13758-bib-0023] Siebenmann, C. , Robach, P. , & Lundby, C. (2017). Regulation of blood volume in lowlanders exposed to high altitude. Journal of Applied Physiology, 123(4), 957–966. 28572493 10.1152/japplphysiol.00118.2017

[eph13758-bib-0024] Sutton, J. R. , Reeves, J. T. , Wagner, P. D. , Groves, B. M. , Cymerman, A. , Malconian, M. K. , Rock, P. B. , Young, P. M. , Walter, S. D. , & Houston, C. S. (1988). Operation Everest II: Oxygen transport during exercise at extreme simulated altitude. Journal of Applied Physiology, 64(4), 1309–1321. 3132445 10.1152/jappl.1988.64.4.1309

[eph13758-bib-0025] Westerterp‐Plantenga, M. S. , Westerterp, K. R. , Rubbens, M. , Verwegen, C. R. T. , Richalet, J.‐P. , & Gardette, B. (1999). Appetite at “high altitude” [Operation Everest III (Comex‐'97)]: A simulated ascent of Mount Everest. Journal of Applied Physiology, 87(1), 391–399. 10409600 10.1152/jappl.1999.87.1.391

